# Leveraging RNA Interference to Impact Insecticide Resistance in the Colorado Potato Beetle, *Leptinotarsa decemlineata*

**DOI:** 10.3390/insects14050418

**Published:** 2023-04-27

**Authors:** Kenan Timani, Pierre Bastarache, Pier Jr Morin

**Affiliations:** Department of Chemistry and Biochemistry, Université de Moncton, 18 Antonine-Maillet Avenue, Moncton, NB E1A 3E9, Canada; ekt7790@umoncton.ca (K.T.); epb6696@umoncton.ca (P.B.)

**Keywords:** Colorado potato beetle, cytochrome P450s, ABC transporters, glutathione S-transferases, insecticide resistance, potato pest, RNA interference

## Abstract

**Simple Summary:**

The Colorado potato beetle is an insect pest known for the substantial damage it can cause on potato crops worldwide. Various strategies, including insecticides, have been deployed to limit its impact, with different levels of success. Insecticide resistance is often observed in this insect, and understanding the factors responsible for this adaptation is important towards the goal of mitigating this challenge. This review will thus highlight key targets associated with insecticide resistance in the Colorado potato beetle and present the potential of RNAi in modulating the susceptibility of this pest towards diverse compounds.

**Abstract:**

The Colorado potato beetle, *Leptinotarsa decemlineata* Say, is a potato pest that can cause important economic losses to the potato industry worldwide. Diverse strategies have been deployed to target this insect such as biological control, crop rotation, and a variety of insecticides. Regarding the latter, this pest has demonstrated impressive abilities to develop resistance against the compounds used to regulate its spread. Substantial work has been conducted to better characterize the molecular signatures underlying this resistance, with the overarching objective of leveraging this information for the development of novel approaches, including RNAi-based techniques, to limit the damage associated with this insect. This review first describes the various strategies utilized to control *L. decemlineata* and highlights different examples of reported cases of resistances against insecticides for this insect. The molecular leads identified as potential players modulating insecticide resistance as well as the growing interest towards the use of RNAi aimed at these leads as part of novel means to control the impact of *L. decemlineata* are described subsequently. Finally, select advantages and limitations of RNAi are addressed to better assess the potential of this technology in the broader context of insecticide resistance for pest management.

## 1. Introduction

Many insects worldwide are known for the significant threat they pose to the agricultural sector. The Colorado potato beetle, *Leptinotarsa decemlineata* Say, is an excellent example of such an insect pest and is known for the substantial damage that it can cause to potato crops. Various means, including the use of multiple insecticides, have been deployed to regulate its spread and to mitigate its impact on the potato industry, yet its ability to develop resistance against these diverse approaches remains remarkable. Significant efforts have thus been directed towards gathering information that would provide a better understanding of the molecular mechanisms that underlie the development of insecticide resistance in this insect in order to better circumvent this challenge. With the goal of presenting key findings associated with insecticide resistance in *L. decemlineata*, this work reviews studies that have highlighted evidence of its resistance towards different compounds and shows key molecular targets that are emerging as potential players in the development of this resistance. It also presents novel avenues, including the use of RNA interference (RNAi)-based approaches, to consider when devising novel strategies aimed at tackling the observed insecticide resistance in this notorious potato pest.

## 2. The Colorado Potato Beetle and Its Control

The Colorado potato beetle is an insect that causes damage to potato crops worldwide and is considered a primary pest for potato plants [1,2]. It also poses a significant threat to tomatoes and eggplants [3]. Substantial plant defoliation is observed when this insect invades a potato field, as notably exemplified by the evidence that states that one single *L. decemlineata* can consume an area equivalent to 40 cm^2^ of potato leaves while in its larval stage [4]. Up to three *L. decemlineata* generations can be generated yearly and a female can produce approximately 800 eggs throughout its life, further supporting the extent of harm that this insect could cause in potato fields [4,5]. The defoliation caused by Colorado potato beetles is associated with important damage to potato crops and production on an annual basis, the extent of which can depend on whether the attacks occur prior to tuber initiation or after [6]. While the impacts underlying potato plant defoliation are often attributable to *L. decemlineata*, it is noteworthy to mention that this insect is not the only culprit responsible for the extensive damage caused to potato crops by insects, as approximately 34% of the losses observed in potatoes on an annual basis result from other insects such as the green peach aphid *Myzus persicae* or the potato leafhopper *Empoasca fabae* to name two [7,8]. Nevertheless, *L. decemlineata* is a key pest for which the damages observed every year in potato fields are attributable. Ultimately, with potatoes being cultivated in approximately 150 countries around the world, considering that the potato is only surpassed by rice and wheat for its global importance [9], strategies to protect this crucial industry from Colorado potato beetles and other threats are essential.

Given its ability to impact potato crops significantly, multiple approaches have been devised over the years to limit the impact of *L. decemlineata*. Several research groups have, in fact, provided excellent overviews of the diverse options deployed to regulate this potato pest [3,10,11,12,13]. For example, the use of biological approaches, such as leveraging known enemies of the targeted pest, including the predatory ground beetle *Pterostichus melanarius* [14] or the green lacewing *Chrysoplerla carnea* [15], has been tested yet with varying levels of efficacy. Sprays consisting of isolates obtained from *Bacillus turingiensis* (Bt) or *Beauveria bassiana* are select examples of additional strategies that have been explored for the control of *L. decemlineata* [16,17]. The use of crop rotation has also been explored and resulted in crop yield increases in select studies [18]. While these options as well as multiple additional alternatives exist to limit the damage caused by this insect pest, the primary strategy for its control remains the use of insecticides. It is estimated that more than hundreds of different compounds have been used to control *L. decemlineata* since the late 19th century [10,19]. Resistance towards these compounds has often developed rapidly as exemplified by the report of *L. decemlineata* resistance against DDT, which developed a little over a decade after it was first tested on this insect [20]. Current compounds in use for *L. decemlineata* control consist of, but are not limited to, diverse chemicals associated with various classes and modes of action, including neonicotinoids or diamides to name two. Unfortunately, as observed with DDT, this insect pest rapidly develops resistance towards these products. It was recently reported that *L. decemlineata* was one of the fastest insect pests capable of developing a resistance against novel compounds, warranting a better understanding of this adaptation [21].

Resistance towards insecticides has thus been explored in *L. decemlineata* by multiple research groups. A significant body of work has been directed at highlighting the resistance of this potato pest against specific compounds from the neonicotinoids class of insecticides including imidacloprid and thiamethoxam. For example, pioneering work performed in adults and larvae sampled in a Long Island population of insects revealed increased resistance towards the former of a value 100.8 times greater than the one observed in insects that were susceptible [22]. Insects obtained from this geographical location displayed the highest levels of imidacloprid tolerance when *L. decemlineata* populations sampled from more than one hundred locations in five countries were evaluated for their response to this compound [23]. A study of *L. decemlineata* populations sampled from diverse locations in the United States and Canada highlighted variations in the extent of imidacloprid resistance, yet they reported a 37-fold resistance to this compound when compared with susceptible insects used as controls [24]. Work conducted in second-instar larvae from different provinces in Canada showed on a given year that up to 45% of the tested populations could display resistance against imidacloprid [25]. The potential for cross-resistance between the neonicotinoids imidacloprid, thiamethoxam, and clothianidin was also raised in this study. It is interesting to note that the temporal assessment of imidacloprid susceptibility during a growing season showed that the response to this compound fluctuates in resistant insects and that increased resistance was observed in the second generation of insects [26]. A study using field populations sampled from various locations in the United States between 1998 and 2010 showed that 80% of the investigated populations had greater LD_50_ values for the neonicotinoid thiamethoxam by the end of that period when compared with the susceptible controls [27]. Examples of resistance against other insecticides, in addition to neonicotinoids, have also been reported. An increase in resistance ratios pertaining to spinosad, a mixture of spinosyns A and D, was observed in *L. decemlineata* populations collected in organically managed fields from eastern New York [28]. Work assessing the response against a panel of 14 insecticides in seven *L. decemlineata* populations collected from China revealed strong resistances in select populations towards different insecticides, including cyhalothrin, deltamethrin, and alpha-cypermethrin to name a few [29]. Finally, it is also worthwhile to point out the recent reports that have explored underlying factors associated with evolution of insecticide resistance in *L. decemlineata*, including how polygenic resistance can provide insights regarding the genomic patterns tied to this resistance [30] as well as how temporal sampling can reveal evidence for selection in genes associated with resistance in select insecticide resistant *L. decemlineata* field populations [31]. Overall, these studies all showcase the ongoing interest in understanding the development of resistance against a variety of insecticides in *L. decemlineata*. Luckily, a clearer picture of the molecular basis underlying such resistances in this potato pest is emerging.

## 3. Molecular Players Associated with Insecticide Resistance in Colorado Potato Beetle

While various leads have emerged for their potential impact on insecticide resistance in *L. decemlineata*, multiple reports have highlighted cytochrome P450s as enzymes of particular interest for such resistances in this insect pest as well as in others [32,33]. Pioneering work in *L. decemlineata* revealed that the expression of select transcripts coding for cytochromes P450s, as determined by RNA-seq and quantitative real-time PCR (qRT-PCR) approaches, was significantly up-regulated in a population of insects showing imidacloprid resistance when compared with a susceptible one [26]. A study notably showed increased levels of *CYP4Q3*, *CYP4Q7*, and *CYP6BQ15* transcripts in *L. decemlineata* displaying imidacloprid resistance. Subsequent RNAi-based knockdown of these targets revealed an impact on imidacloprid response in *L. decemlineata*, for which *CYP4Q3* levels were modulated [34]. In addition to studies showcasing a potential role for cytochrome P450s in imidacloprid response, it is noteworthy to point out that knockdown of *CYP350D1* via double-stranded RNA (dsRNA) injection in adult *L. decemlineata* was associated with a positive impact on chlorantraniliprole response [35]. Another study demonstrated changes in transcripts coding for *CYP4C1*, *CYP4G15*, *CYP6A13*, and *CYP9E2* following clothianidin treatment in *L. decemlineata*, and the injection of dsRNA targeting *CYP9E2* was associated with increased susceptibility towards this compound [36]. It is interesting to note that parallel work identified structural variants in *CYP4g15* as notably exemplified by the significant number of single nucleotide polymorphism outliers associated with this target and suggested a potential contribution of this candidate cytochrome P450 in insecticide resistance [37]. Work aimed at characterizing the molecular impact of fluazinam showed elevated levels of *CYP9Z14v2* transcripts in *L. decemlineata* larvae exposed to the highest concentration investigated [38]. An overview of studies that investigated the differential expression of cytochrome P450s in *L. decemlineata* that were treated to insecticides or that were confirmed resistant to a given compound is shown in Table 1. These results all support the importance of cytochrome P450s for insecticide response in *L. decemlineata* and warrant further assessment of their impact in this potato pest.

In addition to cytochrome P450s, several other enzymes have been identified as potential players in the resistance of *L. decemlineata* to insecticides and are presented in Table 2. For example, the RNAi-based reduction of *Ldace1* expression, a transcript coding for an acetylcholinesterase, was associated with increased susceptibility to chlorpyrifos in *L. decemlineata* [45]. Interestingly, parallel work has explored the structural and regulatory variations associated with this target, as well as *Ldace2*, in two *L. decemlineata* populations [46]. This investigation notably revealed that these populations exhibited differences in susceptibility towards two insecticides and that such a change could be attributable to insecticide target site modifications supporting the ongoing exploration of target site resistance in this potato pest by other groups [47]. A study that leveraged *L. decemlineata* transcriptome and aimed at the carboxylesterase/cholinesterase superfamily notably identified five carboxylesterases that were induced following fipronil exposure, *KM220527*, *KM220530*, *KM220541*, *KM220566*, and *KM220576*, and nine carboxylesterases that responded to cyhalotrin treatments, *KM220527*, *KM220538*, *KM220541*, *KM220542*, *KM220554*, *KM220561*, *KM220564*, *KM220566*, and *KM220578* [48]. Up-regulation of transcripts coding for a glutathione synthetase was reported in two imidacloprid-resistant populations of *L. decemlineata* when contrasted with their expression in a susceptible population [49]. Comparison of the transcriptomes from imidacloprid-resistant *L. decemlineata* with one obtained from susceptible insects demonstrated elevated levels in the former of the transcripts coding for two UDP-glycosyltransferases, *UGT1* and *UGT2*. Subsequent RNAi-based silencing of *UGT2* expression significantly increased the susceptibility of the resistant strain to imidacloprid supporting its potential role in imidacloprid resistance [34]. A recent study showed overexpression of transcripts coding for a peroxidase in an imidacloprid-resistant population of *L. decemlineata* relative to the expression of the same target in a population susceptible to this neonicotinoid [39]. Finally, a significant down-regulation of three transcripts coding for glutathione S-transferases was observed in *L. decemlineata* treated with spinosad for a short period supporting a potential involvement of this additional family of enzymes for insecticide response in this insect [40].

In addition to families of enzymes identified as likely modulators of insecticide resistance in insects, including *L. decemlineata*, other non-enzymatic families of proteins have also been linked to this resistance. ATP-binding cassette (ABC) transporters, transmembrane pumps that can facilitate the cellular export of diverse compounds such as toxins in an ATP-dependent manner, are examples of such targets. ABC transporters have also been positioned as interesting molecular players to leverage for the monitoring of insecticide resistance in various pests [50]. Considering their overarching functions, it is not surprising to find studies that explored ABC transporters within the context of insecticide resistance in *L. decemlineata*. Overexpression of transcripts coding for one ABC transporter from the G subfamily was observed by next-generation sequencing and via qRT-PCR in *L. decemlineata* that displayed imidacloprid resistance when compared with levels measured in a susceptible population [34]. Subsequent RNAi-mediated reduction of its expression in insects using dsRNA-treated potato leaves was not associated with an impact on imidacloprid susceptibility. Reducing the expression of select transcripts coding for ABC transporters in imidacloprid-resistant *L. decemlineata* was associated with increased mortality in insects treated with this neonicotinoid underlying their potential role in such resistance [51]. Knockdown of genes coding for ABC transporters simultaneously or individually caused an increase in imidacloprid-induced mortality in resistant *L. decemlineata*, confirming their contribution to insecticide resistance. Characterization of three members from the B subfamily of ABC transporters, termed *LedMDR1*, *LedMDR2*, and *LedMDR3*, was also conducted in multiple tissues isolated from *L. decemlineata* [52]. Although knockdown of these targets did not lead to an increase in ivermectin susceptibility, assessing the impact of reducing the endogenous levels of these transporters in insects resistant to other compounds, nevertheless, remains of interest to further understand their role, if any, in insecticide response. It is interesting to point out that RNAi-based modulation of *LdABCB1* transcript levels was also linked to a reduction in Cry3Aa toxin-mediated toxicity in *L. decemlineata* larvae [53]. Select studies that have highlighted the importance of ABC transporters for insecticide resistance in *L. decemlineata* are presented in Table 3. Overall, these findings support a closer investigation of the potential impact of ABC transporters on insecticide resistance in this potato pest.

While a consequential amount of work has been performed to better characterize the function of insecticide resistance in the previously mentioned targets in Colorado potato beetles, it is important to highlight additional molecular leads that have been investigated for a similar role in this potato pest. For example, a proteomics-based approach revealed the elevated expression of a C-type lectin in adult *L. decemlineata* that exhibited resistance to imidacloprid and treatments of a susceptible laboratory strain with this compound were associated with elevated levels of C-type lectin in the collected midgut samples, warranting a closer attention to the potential role of this family of proteins in imidacloprid resistance [54]. Expression levels of transcripts coding for select heat shock proteins (HSPs) were modulated in *L. decemlineata* exposed to different stress including temperature and insecticides. Furthermore, changes in *HSP60* transcript levels using dsRNA were associated with substantial mortality in adult insects [55]. It is also important to mention work conducted by multiple teams to better delineate the function of cuticular proteins in *L. decemlineata* within the context of insecticide resistance. A pioneering study showed elevated levels of cuticular protein gene expression levels in *L. decemlineata* resistant to the compound azinphos-methyl [56]. Additional work reported the identification of 175 genes coding for cuticular proteins in *L. decemlineata*, setting the stage for a thorough assessment of the impact of these targets in the response against various insecticides for this insect [57]. Interestingly, a recent study using whole-genome sequencing and transcriptomics information highlighted comparable genetic pathways, but different genes, used by *L. decemlineata* to develop insecticide resistance [58]. Finally, it is noteworthy to mention information stemming from different groups that revealed miRNAs, short non-coding transcripts that can regulate gene expression in a posttranscriptional manner, with potential roles for insecticide resistance and response in *L. decemlineata* [59,60]. Overall, it is clear from the molecular players presented in this section that many leads with a likely relevance for insecticide resistance in *L. decemlineata* have emerged over the past decade. Varying the expression of these targets via diverse strategies could be key to influence such resistance in this potato pest.

## 4. RNAi and Insecticide Resistance

The overarching mechanisms of action associated with RNAi, the use of this technology to silence the expression of molecular targets in insects for various purposes, as well as the challenges associated with such an approach have been reviewed extensively by different research groups [61,62,63,64]. Target silencing via RNAi-based strategies in a given insect can involve the use of dsRNA. The presence of dsRNAs can trigger the modulation of the small interfering RNA (siRNA) pathway and contribute to their subsequent cleavage into siRNA molecules [65]. These fragments can become integral components of the RNA-induced silencing complex, which can facilitate binding to the targeted mRNA, ultimately preventing its translation [66]. This approach thus allows the reduced expression of a given molecular target and holds great promise in the field of insect pest control. In general, much progress has been made towards the optimization of RNAi delivery to make this technology a bona fide tool to control insect pests in an agricultural setting. Significant efforts have been deployed to identify ideal dsRNA packaging systems with dsRNA degradation as well as optimal dsRNA uptake in mind. Such approaches include the use of dsRNA-producing bacterial systems [67], incorporation of dsRNA into nanoparticles [68], as well as the use of plant-based dsRNA delivery systems [69] to name a few. Different RNAi-based products have received approval for commercialization or are in the process of securing it, with the objective of improving crop quality, and have been presented elsewhere [70,71]. Recent examples highlighting the potential of dsRNA specifically for *L. decemlineata* control notably include the successful use of a dsRNA-based insecticide in field and greenhouse trials [72,73]. This insecticide also displayed similar efficiency to other traditional insecticides used against *L. decemlineata*, including chlorantraniliprole and spinosad. In general, RNAi technology appears to hold great promise to control insect pests, and it is not surprising to notice multiple reports showcasing its use against *L. decemlineata*.

Several studies have thus explored the impact of RNAi-mediated knockdown in *L. decemlineata*, either alone or in the context of insecticide resistance. RNAi-based silencing of *STT3b*, *DAD1*, and *GCS1*, three N-glycosylation related genes involved in the early steps of N-glycosylation, resulted in substantial mortality of fourth-stage *L. decemlineata* larvae [74]. Feeding trials in a laboratory setting using dsRNA designed against the *Mesh* gene were proven effective in second- and fourth-stage *L. decemlineata* larvae [75]. Efficiency in field trials was also shown in this study, notably via evidence of significantly greater insect mortality observed in plants treated with dsMesh versus untreated plants. Furthermore, the efficiency of a dsRNA targeting the proteasome subunit beta type-5 was recently shown in a greenhouse trial and was associated with an efficiency comparable to spinosad [72]. A more extensive list that showcases examples of target genes investigated as part of RNAi-based methods and that are associated with *L. decemlineata* control can be found elsewhere [76]. In line with this review, RNAi-based approaches also proved successful in impacting insecticide response in *L. decemlineata*. Pioneering work on the use of RNAi to modulate insecticide susceptibility revealed that the dsRNA-based knockdown of the transcription factor *CncC*, as well as of four cytochrome P450s with relevance to this transcription factor, played a role in imidacloprid resistance [41]. Subsequent work demonstrated that the reduction of genes coding for targets requiring *CncC* for their expression was associated with a greater mortality in imidacloprid-resistant insects, further suggesting a role for this transcription factor in regulating the resistance against this neonicotinoid [51]. The DsRNA-mediated knockdown of genes coding for a cuticular protein, a cytochrome P450, and a glutathione synthetase in second-instar larvae exhibiting imidacloprid resistance could enhance the susceptibility against this compound [77]. Examples of targets potentially underlying insecticide resistance in *L. decemlineata* and explored using dsRNA-based approaches are shown in Table 4. Furthermore, while it is challenging to highlight these studies in the current work, it is relevant to underline that such an approach has seems promising to control a variety of insect pests, including the fall armyworm *Spodoptera frugiperda* [78], the whitefly *Bemisia tabaci* [79], and the emerald ash borer *Agrilus planipennis* [80].

While several advantages are associated with strategies that rely on RNAi-based approaches to overcome insecticide resistance in insect pests, multiple challenges are, nevertheless, linked with their use in field-based applications and have been reviewed elsewhere [63,81,82,83]. The presence and activity of dsRNases in the digestive system of various insects can negatively impact the RNAi-mediated response in insects fed with dsRNAs [84]. Reports have also shown that RNAi efficiency could vary substantially between different populations of insects that were fed the same dsRNA, showcasing intra-species variation in RNAi response [85]. The possibility that the designed dsRNA may regulate the expression of transcripts against which it was not intentionally directed, leading to off-target effects, is also an important aspect to consider with RNAi-based approaches [86]. Virus-mediated production of viral factors capable of influencing select members of the RNAi pathway and, subsequently, impacting dsRNA efficiency has also been reported [87]. The region targeted by the designed dsRNA has been shown to influence its efficiency as observed via assessing the impact of various dsRNAs on *L. decemlineata* larvae’s survival rates and weights [88]. A recent report showcased the development of dsRNA resistance in *L. decemlineata* following dsRNA treatment through non-transgenic foliar delivery, warranting a closer look at the resistance mechanisms underlying the use of dsRNA-based options [89]. Overall, despite inherent challenges associated with dsRNA use as a means to control insect pests, the advantages of this strategy appear to outweigh these hurdles and to support the ongoing efforts deployed at optimizing this approach.

## 5. Outlook

The potential harm that the Colorado potato beetle causes to the potato industry is evident. Strategies have been deployed over the years to control this pest with different levels of success. This insect can adapt itself to fluctuating temperatures and has demonstrated an inherent ability to resist the many insecticides used to control its spread. As exemplified in this review, significant breakthroughs have been made towards the better characterization of the molecular bases underlying the resistances against insecticides in *L. decemlineata*. Leveraging these signatures as part of novel avenues to regulate this potato pest is envisioned. RNAi-based methods are notably appealing, considering the strong response this insect has exhibited when targeted by this strategy. On the other hand, hurdles remain to be cleared as well as important aspects must be clarified regarding the use of large-scale approaches relying on RNAi before its broader use in the agricultural world. These challenges notably include the potential off-target effects linked with RNAi, the development of resistances associated with this strategy, as well as the overall cost of dsRNA production at a sizeable scale to name a few. Nevertheless, with the growing number of mechanistic insights regarding insecticide resistance in *L. decemlineata* that are reported by various groups on an ongoing basis as well as with the increasing understanding of the advantages associated with RNAi, it appears that multiple tools based on this technology are in the development pipeline and could make a tangible difference in the control of this potato pest in the near future.

## Figures and Tables

**Table 1:** Cytochrome P450s with relevance to insecticide response in Colorado potato beetles. Differentially expressed cytochrome P450s identified in *L. decemlineata* either exposed to different compounds or displaying resistance to select compounds. Methods used to quantify changes in cytochrome P450s are also provided. Abbreviations: Control (C), Resistant (R), Susceptible (S), Treated (T). Symbols: ↓ indicates down-regulation and ↑ indicates up-regulation.

Cytochrome P450s	Insecticides	Conditions	Methods	References
^1^ CYP4C1↓, CYP4G57↑, CYP6BJ1↑, CYP6BK17↓, CYP6BQ15↑, CYP6EF1↑, CYP6K1↑, CYP9Z12v1↑	Imidacloprid	R vs. S adults	RNA-seq	[34]
CYP4C1↑, CYP4G15↑, CYP6A13↑, CYP9E2↑ CYP4G15↑ CYP6A23↑	Clothianidin Imidacloprid Spinosad	T vs. C adults	qRT-PCR	[36]
CYP9Z14v2↑	Fluazinam	T vs. C larvae	qRT-PCR	[38]
CYP6K1↑	Imidacloprid	R vs. S adults	qRT-PCR	[39]
CYP6A13↓, CYP6A23↑, CYP6D4↓, CYP12A5↓	Spinosad	T vs. C adults	qRT-PCR	[40]
CYP6BJa/b↑, CYP6BJ1v1↑, CYP9Z25↑, CYP9Z29↑	Imidacloprid	T vs. C cells	qRT-PCR	[41]
CYP4BN13v1↑, CYP4BN15↑, CYP6BH2↑, CYP6BJ1↑, CYP6BQ17↑, CYP6BU1↓, CYP6EG1↑, CYP6EH1↑, CYP6EJ1↑, CYP9Z10↓, CYP12H2↑, CYP12J1↓	Cyhalotrin	T vs. C larvae	qRT-PCR	[42]
^2^ CYP6EH1↑, CYP6EZ1↑, CYP6FA1↑, CYP9A↑, CYP9Ya↑, CYP9Yb↑, CYP9Yc↑, CYP9Z14v1↑, CYP9Z25↑, CYP9Z26↑, CYP9Z28↑, CYP9Z29↑, CYP9Zd↑, CYP9Zi↑, CYP9Zj↑, CYP9Zk↑, CYP4BN13v4↑, CYP4Qb↑, CYP4c↑, CYP4e↑	Imidacloprid	R vs. S adults	qRT-PCR	[43]
CYP6K1↑ CYP4D2↑, CYP6K1↑, CYP49A1↑	Chlorothalonil Imidacloprid	T vs. C adults	RNA-seq	[44]

^1^ Presented are targets that showed absolute log2 fold-changes higher than 5.0. ^2^ Shown are transcripts that displayed increased transcript levels greater than 5.0-fold in resistant versus susceptible insects.

**Table 2:** Additional enzymes of interest for insecticide susceptibility in Colorado potato beetles. Differential expression of transcripts coding for enzymes in *L. decemlineata* treated with or resistant to insecticides. Abbreviations: Control (C), Resistant (R), Susceptible (S), Treated (T). Symbols: ↓ indicates down-regulation and ↑ indicates up-regulation.

Enzymes	Insecticides	Conditions	Methods	References
^1^ Acetylcholin EST1↑, alpha EST↓, carboxyl EST1↑, EST4↑, EST5↑, EST11↓, EST Beta↑, EST FE4↑, GST Sigma 1↑, GST Sigma 2↑, GST1↑, GST7↓, UGT2C1↑, UGT1↑	Imidacloprid	R vs. S adults	RNA-seq	[34]
GST↑, GST1-Like↑	Clothianidin	T vs. C adults	qRT-PCR	[36]
Ldace1↑, Glutathione synthetase↓, UGT1↓	Fluazinam	T vs. C larvae	qRT-PCR	[38]
Lde_KM220566↑, Lde_KM220530↑, Lde_KM220576↑, Lde_KM220527↑, Lde_KM220541↑ Lde_KM220578↑, Lde_KM220566↑, Lde_KM220542↑, Lde_KM220564↑, Lde_KM220561↑, Lde_KM220554↑, Lde_KM220527↑, Lde_KM220538↑, Lde_KM220541↑	Fipronil Cyhalotrin	T vs. C larvae	qRT-PCR	[48]
Glutathione synthetase↑	Imidacloprid	R vs. S adults	RNA-seq	[49]
Peroxidase↑	Imidacloprid	R vs. S adults	qRT-PCR	[39]
GST↓, GST1↓, GST1-Like↓	Spinosad	T vs. C adults	qRT-PCR	[40]
UDP-glucuronsyltransferase 2b7↑, oxidoreductase↑ UDP-glucuronsyltransferase 2b7↑, venom carboxylesterase-6↑	Chlorothalonil Imidacloprid	T vs. C adults	RNA-seq	[44]

^1^ Presented are targets that showed absolute log2 fold-changes higher than 5.0.

**Table 3:** ABC transporters associated with insecticide response in Colorado potato beetles. Studies presenting modulated ABC transporters in *L. decemlineata* susceptible or resistant to select compounds. Methods leveraged to measure changes in ABC transporters are shown. Abbreviations: Resistant (R), Susceptible (S). Symbols: ↓ indicates down-regulation and ↑ indicates up-regulation.

ABC Transporters	Insecticides	Conditions	Methods	References
^1^ ABC-B6↑, ABC-G↑, MRP↓, MRP-1↓, MRP 4-1↑, MRP 4-4↓	Imidacloprid	R vs. S adults	RNA-seq	[34]
ABCH278B↑, ABCH278C↑, ABCG1041A↑	Imidacloprid	R vs. S adults	qRT-PCR	[51]

^1^ Presented are targets that showed absolute log2 fold-changes higher than 3.0.

**Table 4:** dsRNA-based approaches and insecticide susceptibility in Colorado potato beetles. A list of select targets with relevance to insecticide resistance in *L. decemlineata* as investigated in studies that used dsRNA. Means utilized to deliver dsRNA are also presented.

Targets	Insecticides	dsRNA Delivery	References
CYP9E2	Clothianidin	dsRNA injection in adults	[36]
ABCH278B, ABCH278C, ABCG1041A	Imidacloprid	dsRNA feeding in adults	[51]
CncC, CYP6BJa/b, CYP6BJ1v1, CYP9Z25, CYP9Z29	Imidacloprid	dsRNA feeding in adults	[41]
CP18.7, CYP9E2, Glutathione synthetase	Imidacloprid	dsRNA feeding in larvae	[77]

## Data Availability

Not applicable.
